# US public opinion regarding proposed limits on resident physician work hours

**DOI:** 10.1186/1741-7015-8-33

**Published:** 2010-06-01

**Authors:** Alexander B Blum, Farbod Raiszadeh, Sandra Shea, David Mermin, Peter Lurie, Christopher P Landrigan, Charles A Czeisler

**Affiliations:** 1 Department of Health and Evidence Policy, Mount Sinai School of Medicine, New York, NY USA; 2 Division of Cardiology, Department of Medicine, Montefiore Medical Center, Albert Einstein College of Medicine, Bronx, NY, USA; 3 Committee of Interns and Residents, SEIU Healthcare Division, Service Employees International Union, New York, NY, USA; 4 Lake Research Partners, Berkeley, CA, USA; 5 Public Citizen's Health Research Group, Washington, DC, USA; 6 Division of General Pediatrics, Department of Medicine, Children's Hospital Boston, Harvard Medical School, Boston, MA, USA; 7 Harvard Work Hours, Health and Safety Group, Division of Sleep Medicine, Harvard Medical School, Boston, MA, USA; 8 Division of Sleep Medicine, Department of Medicine, Brigham and Women's Hospital, Harvard Medical School, Boston, MA, USA; 9 Current address: US Food and Drug Administration, Silver spring, MD, USA

## Abstract

**Background:**

In both Europe and the US, resident physician work hour reduction has been a source of controversy within academic medicine. In 2008, the Institute of Medicine (IOM) recommended a reduction in resident physician work hours. We sought to assess the American public perspective on this issue.

**Methods:**

We conducted a national survey of 1,200 representative members of the public via random digit telephone dialing in order to describe US public opinion on resident physician work hour regulation, particularly with reference to the IOM recommendations.

**Results:**

Respondents estimated that resident physicians currently work 12.9-h shifts (95% CI 12.5 to 13.3 h) and 58.3-h work weeks (95% CI 57.3 to 59.3 h). They believed the maximum shift duration should be 10.9 h (95% CI 10.6 to 11.3 h) and the maximum work week should be 50 h (95% CI 49.4 to 50.8 h), with 1% approving of shifts lasting >24 h (95% CI 0.6% to 2%). A total of 81% (95% CI 79% to 84%) believed reducing resident physician work hours would be very or somewhat effective in reducing medical errors, and 68% (95% CI 65% to 71%) favored the IOM proposal that resident physicians not work more than 16 h over an alternative IOM proposal permitting 30-h shifts with ≥5 h protected sleep time. In all, 81% believed patients should be informed if a treating resident physician had been working for >24 h and 80% (95% CI 78% to 83%) would then want a different doctor.

**Conclusions:**

The American public overwhelmingly favors discontinuation of the 30-h shifts without protected sleep routinely worked by US resident physicians and strongly supports implementation of restrictions on resident physician work hours that are as strict, or stricter, than those proposed by the IOM. Strong support exists to restrict resident physicians' work to 16 or fewer consecutive hours, similar to current limits in New Zealand, the UK and the rest of Europe.

## Background

Reduction in the work hours of resident physicians (junior doctors) has been the subject of considerable controversy both in America and Europe. Under current regulations of the Accreditation Council for Graduate Medical Education (ACGME), the private body that accredits all US residency programs, resident physicians in the US work extended duration (≥24 h) shifts 1-2 times per week and work 80-88 h per week, on average. Actual work hours are frequently even longer [1-3]. By contrast, under the European Working Time Directive (EWTD), their counterparts in the UK have gone in the past several years from having no restrictions, to having work hours restricted to 72 then 56 then 48 h per week; shifts are limited to a maximum of 13 h consecutively [4]. Despite these differences in work hour limits, academic medical communities on both sides of the Atlantic have engaged in similar debate regarding the potential merits and problems of work hour reduction [5-26]. Critics of work hour reduction cite concerns about the costs of replacement providers, workforce sufficiency, disrupted continuity of care and resident physicians' training experiences [8,27]. Conversely, supporters of reform cite the hazards of extended shifts, which have been shown to induce performance impairment comparable to an increased blood alcohol concentration [28,29], and increase the risk of failures of attention, serious medical errors, fatigue-related preventable adverse events, percutaneous injuries and motor vehicle crashes [2,3,30-35]. Chronic sleep loss, induced by long work weeks, and acute sleep loss, induced by extended duration work shifts, interact synergistically to severely degrade performance, especially at night [36]. A meta-analysis of studies from 959 physicians revealed that following 24 h without sleep, their clinical performance declines to the 7th percentile of their rested performance [37].

In 2007, the US House of Representatives Committee on Energy and Commerce requested the Agency for Healthcare Research and Quality to sponsor an evidence-based assessment of the issue by the Institute of Medicine (IOM) [38], prompted by evidence that 20% of physicians in training admitted making a fatigue-related error that injured a patient (preventable adverse event) and 5% admitted making a fatigue-related error that resulted in the death of a patient (fatal preventable adverse event) during their internship [32,38,39].

In December 2008, following a year-long investigation, the US Institute of Medicine (IOM) released a report, *Resident Duty Hours: Sleep, Supervision and Safety *[40], in which it concluded that scheduling resident physicians to work for more than 16 h consecutively without sleep is unsafe both for patients and resident physicians themselves [40]. The IOM recommended that teaching hospitals either: (1) limit work shifts of resident physicians to 16 h; or (2) permit 30-h shifts, but provide 5 h of protected time for sleep in the hospital after 16 h of work, provided that residents do not admit new patients during the second portion of the shift. The IOM also recommended that: resident physicians be provided at least one 24-h interval off every 7 days, without averaging, and at least one continuous 48-h interval off per month; the work week be limited to 80 h, averaged over 4 weeks, including moonlighting; the ACGME ensure adequate, direct, onsite supervision be provided for residents; the ACGME should enhance monitoring of work hour limits, with additional oversight provided by the Centers for Medicare and Medicaid Services and the Joint Commission (a private sector, not-for-profit organization that accredits US hospitals); and teaching hospitals immediately begin providing transportation home for resident physicians impaired by fatigue.

The ACGME convened a Duty Hours Congress in 2009, at which considerable opposition to the IOM recommendations was voiced, and has since surveyed the opinions of residents, faculty and program directors, commissioned three external reviews of the literature and formed a Duty Hours Task Force. In November 2009, the American Medical Association adopted a policy opposing the IOM recommendation that protected time for sleep be provided during 30-h shifts [41,42].

Despite the vigorousness of the debate, the views of the American public with regard to resident physician work hours, disclosure of resident physician work hours to individual patients, and support for the 2008 IOM recommendations remained largely unknown. Indeed, we are aware of only two prior American public opinion surveys that, tangentially, addressed the issue of resident work hours. In the first, 86% of patients stated that they would be anxious if they knew their surgeon had been awake for 24 h and 70% would ask for a different doctor in that circumstance [43]. In the second study, 74% of respondents listed overwork, stress or fatigue of health professionals as a 'very important cause of medical errors' and 66% felt that reducing the work hours of resident physicians would be very effective in reducing preventable errors [44,45]. To our knowledge, no public surveys on this topic have been conducted elsewhere to date. Understanding the public's views on the IOM's resident work hour recommendations is essential, as it is the safety of the care that they receive while hospitalized that is ultimately at stake. As indicated in the IOM's prior landmark report, *Crossing the Quality Chasm*, a leading aim in efforts to improve the quality of the healthcare system is to better incorporate the beliefs, preferences, and experiences of patients into decisions about the manner in which healthcare is delivered [46].

To better understand the views of the American public on the hours resident physicians currently work and to gauge their reactions to the IOM's proposed limits on resident physician work hours, we administered a public opinion survey to a representative sample of the US population, the most comprehensive survey of public opinion on this issue to date.

## Methods

**Figure 1:**
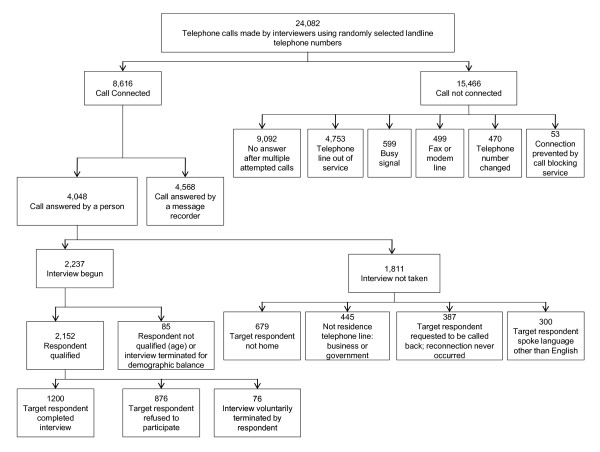
**Flowchart for participation in national US public opinion survey**.

On 17-22 November 2009 and on 21-30 January 2010, 1,200 members of the general American public participated in an 18-min telephone survey (Figure 1) that was conducted by an independent public opinion research firm (Lake Research Partners, Berkeley, CA, USA). Live interviewers provided by McGuire Research Services LLC (Denver, CO, USA) dialed randomly generated landline telephone numbers, stratified by region using area codes and phone exchanges, and either conducted a full interview or noted the reasons for failure to complete.

In all, 24,082 numbers were called, with calling patterns adjusted based on response rates to capture a sample of the US adult population comparable to US Census estimates by region, gender, age and race. 4,048 calls were answered by a person; interviewers then asked to speak to the adult (at least 18 years old) member of that household with the most recent birthday. Of the 4,048 calls answered by a person, 2,152 were determined to be qualified respondents. Of these, 1,200 completed the interview, 876 refused to participate and 76 terminated the call before completing the interview. The response rate (1,200/2,152) was 55.8% (Figure 1). The respondents interviewed between 17-22 November 2009 (N = 800) and the respondents interviewed between 21-30 January 2010 (N = 400) were comparable with respect to both their demographics and their answers to the survey questions, which are combined in the results section.

Participation was strictly voluntary and no personal identifiers were recorded. No financial incentives were provided for participation. Respondents were asked questions about demographic, medical service use, their view on resident physician work hours and the IOM recommendations (see Additional file 1).

The margin of error for the full sample was ± 2.8 percentage points. The data were weighted by gender, race, age, education, and region to ensure that the demographics of the sample matched the US adult population more exactly. Statistical analysis was performed using SAS v.9.1.3 (Cary, NC, USA).

## Results

### Demographic data

The demographic characteristics of the 1,200 study participants were similar to the US adult population (Table 1). The mean age of the survey sample was 47 ± 18.5 years and 51% were female. The participants were well distributed among the four Census Bureau regions, with 91% of participants reporting that they were registered voters. US Census data indicate that 72.1% of voting-age US citizens are registered to vote nationwide [47]; it has been previously documented that survey respondents typically self-report a higher than actual rate of voter registration, which may be due to a social desirability bias [48]. The sample reflected the ethnic and political make-up of the adult population of the US (Table 1). A large proportion of the respondents' households included a health care consumer: 91% reported that someone in their household had seen a doctor in the last year, with 68% reporting three or more visits in the past year (see Additional file 1); 44% reported that someone in their household had been hospitalized in the past 3 years, with 23% reporting that a household member was hospitalized within the past year.

**Table 1:** Characteristics of the survey respondents

Variable	Value	Percentage
Total population	1,200	100

Age, years (mean ± SD)	47 ± 18.5	

Gender, female	612	51

Region:		

Northeast	245	18

Midwest	245	22

South	457	37

West	261	23

Party affiliation:		

Democrat	478	37

Independent	306	27

Republican	306	29

Race/ethnicity:		

White	846	67

Black	122	12

Latino	147	14

Other	85	7

College degree and above	470	36

Hospitalized at least 1 night in last 3 years	528	44

### Resident work hour limits

#### Work hours per shift

Respondents estimated that resident physicians currently work an average shift duration of 12.9-h shifts (95% CI 12.5 to 13.3 h) (for detailed responses, see Table 2). Among the respondents, 82.2% (95% CI 80% to 84.5%) estimated that resident physicians currently average 16 h or less per shift; 11.6% (95% CI 9.7% to 13.6%) estimated the average to be longer than 16 h; and 2% estimated the average to be longer than 24 h. When asked the maximum number of hours resident physicians should work on any given shift (Table 2), responses averaged 10.9 h (95% CI 10.6 to 11.3 h), with 90% (95% CI 89% to 92%) setting the maximum shift duration at 16 h or less. In fact, 85% of the respondents set the maximum shift duration at 12 h or less consecutively (95% CI 82% to 87%). A total of 4% (95% CI 3% to 5.6%) of the respondents set the maximum shift duration at more than 16 h, and 1% (95% CI 0.6% to 2%) set the limit at more than 24 h.

**Table 2:** Beliefs of survey respondents regarding current resident work hours and opinions about appropriate work hours

	Average hours respondents thought residents DO work (95% CI)	Maximum hours respondents believed residents SHOULD work (95% CI)
Duration of individual work shifts (hours):		

8 or less	19 (17 to 22)	32 (29 to 34)

9-12	49 (46 to 52)	53 (50 to 56)

13-18	18 (15 to 20)	6 (5 to 8)

19-23	3 (2 to 5)	1 (0.8 to 2)

≥24	5 (4 to 7)	2 (1 to 3)

Don't know	6 (5 to 7)	5 (4 to 7)

Weekly resident work hours:		

40 or less	21 (18 to 23)	35 (32 to 38)

41-59	16 (14 to 19)	29 (26 to 31)

60-79	31 (28 to 34)	22 (20 to 25)

≥80	18 (16 to 20)	5 (4 to 7)

Don't know	14 (12 to 16)	9 (7 to 10)

#### Weekly work hours

Respondents estimated that resident physicians currently average 58.3 h (95% CI 57.1 to 59.5 h) of work per week (Table 2). Among the respondents, 67% (95% CI 65% to 71%) thought that resident physicians currently average less than 80 h of work per week. The maximum number of hours per week that respondents believed resident physicians should be allowed to work averaged 50 h (95% CI 49.4 to 50.8 h), with 86% (95% CI 84% to 88%) setting the weekly maximum at less than 80 h and 5.5% (95% CI 4% to 7%) at 80 h or more per week.

#### Resident work hours and medical errors

More than four out of five respondents believed that reducing resident work hours would be 'effective' or 'somewhat effective' in reducing medical errors (Table 3). When stratified by political party, the support for this statement was maintained across political party lines.

**Table 3:** Support for selected resident work hour reforms and related beliefs, stratified by political party affiliation, geographic region and race

	Believe lower hours results in reduced medical errors	Supports a cap of 80 h a week, maximum shift of 16 h and 1 day off per week	Favors maximum shift duration of 16 h over 30-h shift with 5 h protected sleep time	Believes that patient should be informed if resident >24 h awake
Total population	81 (79-84)	80 (77-82)	68 (65-71)	81 (79-83)

By political party affiliation:				

Democratic	86 (83-89)	86 (83-89)	72(68-76)	85 (82-88)

Republican	77 (72-82)	76 (71-81)	66 (61-72)	78 (73-83)

Independent	80 (75-85)	76 (71-81)	64 (58-69)	80 (75-84)

By region:				

Northeast	85 (81-90)	81 (75-86)	63 (56-70)	78 (72-83)

Midwest	80 (75-86)	78 (72-83)	69 (63-75)	82 (77-87)

South	80 (76-84)	81 (77-85)	71 (66-75)	81 (77-84)

West	81 (76-86)	79 (74-84)	67 (61-73)	83 (78-88)

By race:				

White	81 (78-84)	78 (75-81)	70 (67-73)	80 (78-83)

Black	81 (74-88)	86 (79-92)	61 (52-71)	88 (82-94)

Latino	81 (74-88)	86 (80-92)	68 (60-76)	80 (73-87)

Column 1 question read: 'Thinking about ways to reduce the frequency of medical errors at hospitals, how effective do you think it would be to reduce the hours worked by medical residents at hospitals - would reducing the hours worked by medical residents be a very effective, somewhat effective, not very effective, or not at all effective in reducing the frequency of medical errors at hospitals?'.

Column 2 question read: 'As you may know, doctors who work as medical residents in hospitals are required to work ≥80 h a week, which can include shifts of up to 30 hours without sleep. Now I would like to read you a proposal that would change those work shift requirements for medical residents. Under this proposal: Work hours would be capped at no more than 80 hours in any single week. Shifts would be capped at a maximum of 16 hours. Medical residents would have at least 5 days off per month, including at least one 24 hour period per week and one 48 hour period per month. Would you favor or oppose this proposal, or are you undecided?'.

Column 3 question read: 'Thinking of the proposal I just read you, imagine you had two versions of the proposal. All elements would remain the same, but with one key difference. One version would say: 'No medical resident is allowed to work more than 16 hours per shift' and the other version would instead say 'Medical residents' shifts are capped at 30 hours in length. During this time, they are allowed a 5-hour nap, and they are not permitted to admit new patients after the first 16 hours'.

Column 4 question read: 'Do you think patients should be informed if a medical resident who is treating them has been working for more than 24 hours?'.

### Public opinions on resident physician work hour regulation

When asked about specific IOM proposal packages, four out of five respondents supported a proposal that included limiting the duration of individual work shifts to 16 h, capping weekly work hours at a maximum of 80 h in any single week and ensuring that medical residents have at least 1 day off per week (Table 3). Of note, 67% of the subgroup of respondents who were health care workers supported this proposal, as did 83% of the subgroup who were health care workers' family members. Among the 20% of the overall respondents who withheld their support for this proposal (opposed or undecided), 44% did so because they thought that the proposal did not go far enough in limiting resident physician work hours. Thus, 89% either supported a proposal with this or stricter work hour limits.

When asked about individual components of the IOM recommendations (Table 4), three-quarters or more of respondents: (1) supported the IOM-proposed 16 h per shift limitation; (2) supported the alternative IOM recommendation requiring that at least 5 h of protected sleep be provided during a 30-h shift; (3) supported the IOM proposal that work hours be capped at no more than 80 h in any single week; (4) supported the IOM proposal that hospitals be required to provide safe transportation home, such as a taxi or public transit ticket, for residents too fatigued to drive home safely; and (5) did not believe that resident physicians should be allowed to work jobs outside of the hospital in their off hours during their training (Table 4). Two-thirds of respondents favored a 16-h maximum shift duration over the alternative IOM recommendation requiring 5 h of protected time for sleep. More than 90% of respondents supported the establishment of strict rules to ensure that medical residents are provided with direct, on-site supervision by more experienced doctors (Table 4).

**Table 4:** US public opinion regarding individual components of Institute of Medicine (IOM) recommendations for resident physician work hour limits

Individual components of a proposal that would change the work shift requirements for medical residents	Support* (percentage of respondents)	Oppose* (percentage of respondents)	Neutral* (percentage of respondents)	Don't know* (percentage of respondents)	Mean rating (95% CI)
Residents would not be allowed to work more than 16 h per shift in patient care	82 (80-84)	10 (8-12)	7 (5-9)	1 (0.4-2)	8.2 (8.0 to 8.4)

Those working a 30-h shift would have at least 5 h of protected sleep time between the hours of 10 pm and 8 am	79 (76-81)	11 (9-13)	8 (7-10)	2 (1-3)	7.9 (7.7 to 8.1)

Work hours would be capped at no more than 80 h in any single week	78 (76-81)	14 (12-16)	6 (4-7)	2 (1-3)	7.9 (7.7 to 8.1)

Medical residents would have at least 5 days off per month, including at least one 24-h period per week and one 48-h period per month.	86 (84-88)	6 (5-8)	7 (5-8)	1 (0.4-2)	8.5 (8.3 to 8.6)

Hospitals would have to provide safe transportation home, such as a taxi or public transit ticket, for residents too fatigued to drive home safely.	77 (74-79)	13 (11-15)	10 (8-12)	0.8 (0.3-1.2)	7.8 (7.6 to 8.0)

Strict rules would be established to ensure that medical residents are provided with direct, on-site supervision by more experienced doctors	91 (89-93)	3 (2-4)	5 (3-6)	1 (0.4-2)	8.9 (8.8 to 9.1)

Medical residents would not be allowed to moonlight, or work jobs outside of the hospital in their off hours.	75 (72-78)	14 (12-16)	9 (7-10)	3 (2-4)	7.8 (7.6 to 8.0)

*Respondents were instructed as follows: 'For each one [question], please rate your support for that item on a scale of 0 to 10, where 10 means you strongly support this component, and 0 means you strongly oppose this component, and you can choose any number in between'. Responses from 6-10 are reported under 'Support'; responses from 0-4 are reported under 'Oppose'; responses of 5 are reported under 'Neutral'. Mean rating is the average of all scores, excluding 'Don't know'.

### Regulatory enforcement

Among the respondents, 49% (95% CI 46% to 52%) favored letting the ACGME be responsible for regulating the hours worked by medical residents with oversight by a federal health agency, whereas 37% (95% CI 34% to 40%) favored letting the ACGME alone be responsible for such regulation. Support for letting the ACGME alone have this responsibility varied by political party affiliation: Democrats, 28%, Republicans, 47%, and Independents, 40%.

### Patient reactions to extended resident physician work shifts

Among the respondents, more than four out of five respondents believed that "patients should be informed if a medical resident who is treating them has been working for more than 24 hours" (Table 3). If they learned that their doctor had been awake for more than 24 h, 85% (95% CI 82% to 87%) reported that they would "feel anxious about the safety of [their] medical care" and 80% (95% CI 78% to 83%) would "want to be treated by a different doctor" (Additional file 1: Table 4).

## Discussion

Only 1% of the American public believes that resident physicians should be allowed to work more than 24 h consecutively. This judgment contrasts with current ACGME policy, which authorizes work shifts of 30 h consecutively without sleep twice each week. Most resident physicians routinely work shifts of this duration [1,2]. As it is the public that funds both residency training (through the Centers for Medicare and Medicaid Services) and healthcare costs, and the public that ultimately suffers the potential safety consequences of healthcare provider impairment, members of the public should from an ethical standpoint have the right to make voluntary and informed health care decisions regarding the care that they receive [46]. The stark discrepancy between the views of the public and current practices regarding resident work hours is therefore of considerable concern. Most resident physicians routinely work shifts exceeding those that the public believes to be safe [1,2]; there is considerable evidence that the public's concerns regarding the patient safety consequences of extended duration shifts and provider sleep deprivation are justified [23,29,31,32,37,49,50]. We found that 89% of the public supports work hour limits that go beyond the IOM recommendations, including a requirement for a shift duration of 16 h or less, as has been in place for resident physicians in New Zealand for 20 years [51]; 85% support an even more stringent 12-h limit, similar to Europe's 13-h limit [52]. On average, the American public supports a 50-h work week limit, slightly higher than the 48-h limit in Europe, but considerably less than the ACGME's 80-h average weekly limit.

Public support was very strong for each of the core components of the IOM recommendations, ranging from 75 to 91%, and held true across political party lines, regions, racial/ethnic groups and gender. When asked to choose, nearly two-thirds (64%) preferred the IOM recommendation that resident physicians not be allowed to work more than 16 h per shift in patient care to the IOM alternative that resident physicians working a 30-h shift be provided at least 5 h of protected sleep time. Two-thirds of the American public believe that resident physicians should not work more than 60 h per week and 86% believe that resident physicians should not work more than 80 h in any week.

Substantial majorities believe reductions in resident work hours would be effective in reducing medical errors, a belief with empirical support [31,32], and that patients should be informed if their treating resident physician had worked for more than 24 h. Support for this disclosure requirement held across political party lines, regions, racial/ethnic group and gender. Survey respondents claimed that they would act on such disclosure: 80% stated that they would request another physician if their physician was working longer than 24 h, an increase from the 70% figure reported in 2002 [43]. Whether or not patients would do so cannot be determined from this study; indeed, patients may not be in a position to refuse care on a case by case basis, which makes determining their views on this issue beforehand and developing appropriate policies in response all the more important. Incorporating the voice of the patient in health care decisions is necessary to respect the ethical principle of autonomy. Patients have the right to guide decisions regarding their care, and should be the ultimate arbiters of what care they receive [3,53].

The public estimate of the average weekly work hours of resident physicians is remarkably accurate [54]. Yet most Americans believe that the average work shift of resident physicians is currently less than 12 h in duration, with only 2% estimating the average shift to be greater than 24 h, perhaps due to the well publicized work hour limits that the ACGME implemented in 2003. Given that the public appears to be unaware that the ACGME set the work shift limit for resident physicians at 30 h consecutively in 2003, a duration that raises safety concerns for 85% of respondents, the failure to disclose whenever a treating physician has been working more than 24 h in the face of the now documented assertion by patients that most would seek alternate medical care on the basis of that information raises serious ethical and policy concerns [3,53].

The public plays a large role in funding the training of physicians; Medicare funding for graduate medical education was nearly US $10 billion in 2008 [55]. The IOM report estimated that an additional $1.7 billion would be needed to fund its recommendations. However, if preventable adverse events were reduced by 11.3%, a reduction well within that observed in one study in the intensive care unit [31], the additional expenditures would be recovered by society [56].

Our study had a number of limitations. First, our response rate was 55.8%. It is thus possible that our population differs from the general population. However, this response rate is typical of rigorously conducted public social science polls, which average 50% [57]. Interviewers did not reveal the topic of the survey before determining whether or not the respondent was willing to participate. Rigorously conducted public opinion surveys with a 50% response rate reached a sample that, though somewhat younger and more educated than the general population, was generally representative of the US population [57,58], To account for this potential bias, we adjusted dialing patterns to ensure that the age distribution of our survey population was representative of the US population, and weighted the data analysis by gender, race, age, education, and region to match the US adult population more exactly. Since the makeup of our sample closely reflected that of the US adult population and there were minimal differences in responses to the survey questions with respect to age, gender, geographic region and race/ethnicity, the results of both the weighted and unweighted data analysis were comparable. Moreover, the Pew Research Center only found small differences between responses from a standard survey, with a typical response rate of 30%, and responses from a rigorous survey with a response rate of 50%, including responses from those interviewees who were the hardest to reach. This is consistent with the comparability in our survey results between the first 800 respondents interviewed in November 2009 and the final 400 respondents interviewed 8 weeks later [57,58]. Finally, the definitive results of this survey are such that it is highly unlikely that an even larger sample would yield substantially different results.

A second limitation was that we relied on landline telephone numbers. Those who maintain landlines may differ from those with only mobile telephones. However, a representative geographic distribution of residences is difficult to achieve using mobile telephone numbers, which often remain unchanged after relocation. Therefore, most public opinion surveys use landlines only. To avoid potential demographic biases related to the use of landlines, calling patterns were adjusted to capture a representative demographic population. A third limitation is that the interviews were conducted solely in English; however, 14% of our respondents identified themselves as Latino, which is comparable with that observed in the US Current Population Survey [57].

## Conclusions

The IOM recommendations represent one of the first efforts to substantively reform resident work hours initiated by the leadership of academic medicine, rather than by legislative bodies. The majority of the American public endorses restrictions on resident work hours that are as stringent as or even more stringent than those put forth by the IOM and endorsed by a coalition of more than 40 public interest and patient safety groups [59]. Our data indicate that only 1% of the American public approves of the 30-h shifts currently authorized by the ACGME for residents working in teaching hospitals. Most respondents also support the addition of federal oversight to the role of the ACGME in regulating resident work hours, as recommended by the IOM. Furthermore, most respondents claim they would request a different doctor if their treating physician were awake more than 24 h. Honoring such requests could have a profound impact on the American health care delivery system, given that the 108,000 resident physicians provide much of the direct medical care in US teaching hospitals [60] and that teaching hospitals, which represent 22% of all US hospitals, provide care for 53% of all hospitalized patients and charity care for 71% of all hospitalized patients who lack adequate medical insurance and are unable to pay for their own care [61]. As the public ultimately bears the consequences of decisions affecting the safe delivery of healthcare, their opinions should be seriously considered by policy makers who regulate resident physician work hours. In other countries struggling to balance competing concerns about resident physician work hour reform, the perspectives of the public could likewise be important to consider.

## Competing interests

The nationwide survey was sponsored by the Committee of Interns and Residents, which is the largest union of resident physicians in the US and is affiliated with the Service Employees International Union, by Public Citizen, a national non-profit consumer advocacy organization, and by the American Medical Student Association (AMSA), a student-governed, national organization committed to representing the concerns of physicians in training in the US. The Committee of Interns and Residents contracted with an independent public opinion survey firm, Lake Research Partners, to design and analyze the survey, and did not impose any limitations on the publication of the study's results. All authors have completed the Unified Competing Interest form at http://www.icmje.org/coi_disclosure.pdf (available on request from the corresponding author). ABB is an employee of Mount Sinai School of Medicine and a paid consultant to the Committee of Interns and Residents of the Service Employees International Union (CIR/SEIU). FR is an employee of Montefiore Medical Center and is President of CIR/SEIU. SS is an employee of CIR/SEIU. DM is an employee of Lake Research Partners, an independent public opinion research firm that was provided a contract for services provided in designing, conducting and analyzing the nationwide survey reported herein. PL was previously an employee of Public Citizen. CPL is an employee of Children's Hospital Boston and of Brigham and Women's Hospital, a subsidiary of Partners HealthCare, and is on the faculty of Harvard Medical School. CPL also reports serving as a paid consultant to the District Health Boards of New Zealand, providing recommendation on how to improve the scheduling and working conditions for junior doctors in New Zealand; Vital Issues in Medicine, developing an educational course for physicians on Shift Work Disorder (supported by an unrestricted educational grant from Cephalon Inc. to Vital Issues in Medicine); and AXDev, to assist in the development of a study of Shift Work Disorder (supported by an unrestricted research grant from Cephalon Inc. to AXDev). In addition, CPL reports receiving monetary awards, honoraria, and travel reimbursement from multiple academic and professional organizations for delivering lectures on sleep deprivation and safety. CAC is an employee of Brigham and Women's Hospital, a subsidiary of Partners HealthCare, which employs more than 1,000 resident physicians, and is on the faculty of Harvard Medical School. CAC has received consulting fees from or served as a paid member of scientific advisory boards for: Actelion, Ltd; Bombardier, Inc.; Boston Celtics; Cephalon, Inc.; Columbia River Bar Pilots, Delta Airlines; Eli Lilly and Co.; Fedex Kinko's; Federal Motor Carrier Safety Administration (FMCSA), US Department of Transportation; Fusion Medical Education, LLC; Garda Síochána Inspectorate (Dublin, Ireland); Hypnion, Inc. (acquired by Eli Lilly and Co. in April 2007); Global Ground Support; Johnson & Johnson; Koninklijke Philips Electronics, NV; Minnesota Timberwolves; Morgan Stanley; Philips Respironics, Inc.; Portland Trail Blazers; Sanofi-Aventis Group; Sepracor, Inc.; Sleep Multimedia, Inc.; Sleep Research Society (for which CAC served as president); Somnus Therapeutics, Inc.; Takeda Pharmaceuticals; Vanda Pharmaceuticals, Inc.; Vital Issues in Medicine; Warburg-Pincus and Zeo Inc. CAC owns an equity interest in Lifetrac, Inc.; Somnus Therapeutics, Inc.; Vanda Pharmaceuticals, Inc., and Zeo Inc., and received royalties from McGraw Hill, the New York Times and Penguin Press. CAC has received lecture fees from the Accreditation Council of Graduate Medical Education; Alfresa; the American Academy of Allergy, Asthma and Immunology Program Directors; American Physiological Society; Association of University Anesthesiologists; Baylor College of Medicine; Beth-Israel Deaconess Medical Center; Brown Medical School/Rhode Island Hospital; Cephalon, Inc.; Clinical Excellence Commission (Australia); Dalhousie University; Duke University Medical Center; Harvard School of Public Health, Harvard University; Institute of Sleep Health Promotion (NPO); London Deanery; Morehouse School of Medicine; Mount Sinai School of Medicine; National Emergency Training Center; National Institutes of Health; North East Sleep Society; Osaka University School of Medicine; Partners HealthCare, Inc.; Sanofi-Aventis, Inc.; St Lukes Roosevelt Hospital; Takeda; Tanabe Seiyaku Co., Ltd; Tokyo Electric Power Company (TEPCO); University of Michigan; University of Pennsylvania; University of Pittsburgh; University of Tsukuba; University of Virginia Medical School; University of Washington Medical Center; University of Wisconsin Medical School; World Federation of Sleep Research and Sleep Medicine Societies. CAC has also received research prizes with monetary awards from the American Academy of Sleep Medicine; American Clinical and Climatological Association; Association for Patient-Oriented Research; National Institute for Occupational Safety and Health; National Sleep Foundation; and Sleep Research Society; clinical trial research contracts from Cephalon, Inc., Merck & Co., Inc., and Pfizer, Inc.; an investigator-initiated research grant from Cephalon, Inc.; and his research laboratory at the Brigham and Women's Hospital has received unrestricted research and education funds and/or support for research expenses from Cephalon, Inc., Koninklijke Philips Electronics, NV, ResMed, and the Brigham and Women's Hospital. The Harvard Medical School Division of Sleep Medicine (HMS/DSM), which CAC directs, has received unrestricted research and educational gifts and endowment funds from: Boehringer Ingelheim Pharmaceuticals, Inc., Cephalon, Inc., George H Kidder, Gerald McGinnis, GlaxoSmithKline, Herbert Lee, Hypnion, Jazz Pharmaceuticals, Jordan's Furniture, Merck & Co., Inc., Peter C Farrell, Pfizer, ResMed, Respironics, Inc., Sanofi-Aventis, Inc., Sealy, Inc., Sepracor, Inc., Simmons, Sleep Health Centers LLC, Spring Aire, Takeda Pharmaceuticals and Tempur-Pedic. The HMS/DSM has received gifts from many outside organizations and individuals including: Axon Sleep Research Laboratories, Inc., Boehringer Ingelheim Pharmaceuticals, Inc., Catalyst Group, Cephalon, Inc., Clarus Ventures, Eli Lilly and Co., Farrell Family Foundation, Fisher & Paykel Healthcare Corporation, George H Kidder, GlaxoSmithKline, Hypnion, Inc., Jordan's Furniture, Merck Research Laboratories, Park Place Corporation, Respironics, Inc., Sanofi-Aventis, Inc., Select Comfort Corporation, Sepracor, Inc., Sleep Health Centers LLC, Takeda Pharmaceuticals, Tempur-Pedic Medical Division, Total Sleep Holdings, Vanda Pharmaceuticals, Inc. The HMS/DSM Sleep and Health Education Program has received Educational Grant funding from Cephalon, Inc., Takeda Pharmaceuticals, Sanofi-Aventis, Inc. and Sepracor, Inc. CAC is the incumbent of an endowed professorship provided to Harvard University by Cephalon, Inc. and holds a number of process patents in the field of sleep/circadian rhythms (for example, photic resetting of the human circadian pacemaker). Since 1985, CAC has also served as an expert witness on various legal cases related to sleep and/or circadian rhythms.

## Authors' contributions

ABB, SS, DM, PL, CPL, and CAC conceived of and created the study design, and together with FR designed the survey instrument. SS and PL obtained funding for the project. DM, CPL and CAC supervised the overall conduct of the study. DM oversaw survey data acquisition and data analysis through Lake Research Partners. ABB, FR, CPL and CAC prepared the first draft of the manuscript. All authors contributed to the content of the manuscript (including the authors employed by the study's sponsors) and to the design and conduct of the study; the analysis and interpretation of the data; and the preparation of the manuscript. All authors had full access to the statistical reports and data tables in the study. FR performed the statistical analyses reported in the article. All authors read and approved the final manuscript.

## Author information

ABB is a recipient of a Ruth L. Kirschstein National Research Service Award. ABB is currently a Health and Evidence Policy Fellow at Mount Sinai School of Medicine and is matriculated in the Masters in Public Health Program at The Johns Hopkins Bloomberg School of Public Health. ABB is a pediatrician who trained at University of California Los Angeles. He also serves as a consultant to CIR.

FR is currently a fellow in cardiovascular medicine at Montefiore Medical Center, the University Hospital of Albert Einstein College of Medicine. He holds board certification in internal medicine, having completed residency training at St Luke's Roosevelt Hospital Center, an affiliate of Columbia University College of Physicians and Surgeons. He also holds a PhD degree from Cornell University, an MD degree from Tehran University of Medical Sciences, and is President of CIR.

SS serves as policy director for the Committee of Interns and Residents/SEIU, a national union of resident physicians with 13,000 members in more than 50 public and private teaching hospitals in Massachusetts, New York, New Jersey, District of Columbia, Florida, New Mexico and California. SS is also the Program Director of the non-profit CIR Policy and Education Initiative. SS has more than 30 years of experience with medical residency training issues, with a consistent focus on resident work hours, and has organized two national conferences on the design of resident work to enhance patient and physician safety, cosponsored by CIR and the American Medical Association Resident Fellow Section.

DM is a partner at Lake Research Partners (LRP) and heads the firm's Bay Area office. LRP is the national research firm established in 1995 by Celinda Lake, one of the country's leading political strategists. LRP advises dozens of incumbents and challengers at all levels of the electoral process, as well as a wide range of non-partisan advocacy organizations, foundations, and labor unions. LRP has nationally recognized expertise on women voters, health care issues, the environment, and the labor movement, and conducts the bipartisan Battleground Poll, a widely respected barometer of national political trends. LRP has offices in Washington, DC and Berkeley, CA. Formerly, DM worked at The Harwood Group, a non-partisan public issues research and consulting firm, and for the Health Care Financing Administration.

PL is Senior Advisor in the Office of Policy, Planning and Budget in the Office of the Commissioner at the Food and Drug Administration. The views presented here do not represent those of the FDA or the US government. Formerly, PL was Deputy Director of Public Citizen's Health Research Group in Washington, DC, and on the faculties of the University of California, San Francisco and the University of Michigan.

CPL is director of Sleep and Patient Safety at the Brigham and Women's Hospital, Assistant Professor of Pediatrics and of Medicine at Harvard Medical School, a pediatric hospitalist at Children's Hospital Boston and a health services researcher with 12 years' experience studying the quality of inpatient care, patient safety, and the effects of healthcare providers' work hours and sleep deprivation on safety. His research has evaluated the efficiency and quality of care in pediatric hospitalist systems, as well as the safety of hospital care. CPL helped design and conduct some of the first studies to determine rates of medication errors in pediatrics, and over the past several years he has led a series of studies evaluating the relationship between resident sleep deprivation and patient safety; resident depression and patient safety; the effectiveness of computerized order entry systems; and the relationship between resident handoffs and error. CPL is an Executive Council Member and Immediate Past Chair of the Pediatric Research in Inpatient Settings (PRIS) Research Network, a collaboration of over 300 hospitalists who are beginning to study quality and variation in the care of hospitalized children, with the goal of developing and disseminating improvements.

CAC is Director of the Division of Sleep Medicine and the Harvard Work Hours, Health and Safety Group, and the Baldino Professor of Sleep Medicine at the Harvard Medical School; and is Chief of the Division of Sleep Medicine in the Department of Medicine at the Brigham and Women's Hospital. CAC serves as a member of the Steering Committees of the Sleep Research Network and the Academic Alliance for Sleep Research; is a member of the National Center for Sleep Disorders Research Advisory Board of the National Heart Lung and Blood Institute; was President of the Sleep Research Society; and served as a member of the Drowsy Driving Commission for the Commonwealth of Massachusetts. CAC has three decades of experience conducting translational research in circadian physiology, sleep science and sleep medicine, and pioneered the application of sleep and circadian physiology and pathology to occupational medicine. In recognition for his research on the consequences of resident physician work hours, CAC has been inducted as an Honorary Fellow of the Royal College of Physicians, London (FRCP); has received the National Institute of Occupational Safety and Health (NIOSH) Director's Award for the most meritorious research supported by NIOSH in 2006; and will receive the Senator Mark O Hatfield Public Policy Award from the American Academy of Sleep Medicine in 2010.

## Pre-publication history

The pre-publication history for this paper can be accessed here:

http://www.biomedcentral.com/1741-7015/8/33/prepub

## Supplementary Material

Additional file 1 **Supplementary material**. Supplementary methods and supplementary tables 1-4. Click here for file
