# Supplementary material for: US public opinion regarding proposed limits on resident physician work hours
Source: BMC Med. 2010 Jun 1;8:33. doi: 10.1186/1741-7015-8-33 (PMC2901227; doi:10.1186/1741-7015-8-33)
Supplement: Additional file 1 — Supplementary material. Supplementary methods and supplementary tables 1-4. [file 1741-7015-8-33-S1.PDF]

# Supplemental online material:

**Supplemental Methods**

Lake Research Partners designed the survey instrument and contracted with McGuire Research Services LLC to administer the survey.  McGuire Research Services LLC employed trained, professional interviewers, with a minimum supervisor-to-interviewer ratio of 1:10, and conducted callback verification on 10% of the calls to ensure accuracy and precise demographic representation.  Some interviews were terminated for demographic balance in terms of race and age; when this happened a substitute interview was conducted to capture a representative sample. Lake Research Partners monitored a random selection of interviews for quality control.

To accomplish the highest possible response rates, all initial incomplete calls were stored in queues and recalled at prescribed times several hours or the next day after the initial call. Return calls were also attempted with different interviewers to increase the likelihood of a completed interview. Interviewers redialed telephone numbers in the queue when the calls were categorized as: no answer, busy signal or other non-contacts repeatedly, until someone was reached or it was learned that the telephone number was out of service. When a qualified respondent answered the telephone but requested to be called back later, interviewers returned such calls until the full sample had been reached.

Survey interviews were begun with the following script:  “Hello. This is (caller name). I'm calling from National Opinion Surveys. We are conducting a public opinion survey and I would like to ask you some questions. I am not selling anything, and I will not ask for a contribution. Could I please speak with the (MALE/FEMALE) in your household, 18 years or older, who celebrated a birthday most recently?”

Initial survey questions concerned the participants’ own use of medical services, their status as a registered voter, and their political party affiliation, while the final questions concerned

demographic characteristics. Respondents were asked how many hours per week and per shift they thought resident physicians currently work and how many they believed residents should work. Follow-up questions addressed the IOM recommendations on resident work-hour reform. Participants were asked if they supported individual components of the IOM recommendations as well as a proposal package that included four of the major IOM recommendations. Those who did not favor the proposal package were asked if they withheld support because the package went too far or because it did not go far enough to restrict resident work hours.

The survey also included questions about medical errors related to resident fatigue and the need for individual disclosure of resident-physician work hours to patients. Respondents were also questioned about their confidence in the care provided by a physician who had been working in the hospital for 24 hours. Respondents were asked if they thought the ACGME should act alone in regulating work hours or if it should do so "with oversight by a federal health agency." The ACGME was described to respondents as a "private sector, non-profit group made up of doctor and hospital organizations [that] oversees all medical resident training programs."

Each survey response was given a weight of slightly greater or less than 1.0 to compensate for slight overrepresentation or underrepresentation of certain groups within the sample. For example, if the U.S. adult population was 51.0% female and the unweighted sample was 50% female, the female respondents in the sample would be given a weight of 1.02 and the male respondents would be given a weight of 0.98. Ordinal responses were dichotomized, unless otherwise reported.

**Supplemental Table 1.** Opinion of survey respondents on perceived maximum weekly work hours and maximum shift hours, unweighted results.

| | | Average hours respondents thought residents DO work<br><br>Number (percent) | Maximum hours respondents believed residents SHOULD work |
|---|---|---|---|
| Weekly resident work hours | | | |
| | 40 or less | 233 (19) | 427 (36) |
| | 41-59 | 188 (16) | 343 (29) |
| | 60-79 | 375 (31) | 261 (22) |
| | 80+ | 227 (19) | 60 (5) |
| | Don't know | 177 (15) | 109 (9) |
| Length of individual shifts | | | |
| | 8 or less | 224 (19) | 388 (32) |
| | 9-12 | 584 (49) | 630 (53) |
| | 13-18 | 212 (18) | 71 (6) |
| | 19-23 | 39 (3) | 17 (1) |
| | 24+ | 64 (5) | 25 (2) |
| | Don't know | 77 (6) | 69 (6) |

**Supplemental Table 2:** Support for resident work hours reform in general and for specific aspects of the reform stratified by political party affiliation, geographic region, race, and health care affiliation, unweighted analysis.

| | Believe lower hours results in decreased errors | Supports a cap of 80 hours a week, maximum shift of 16 hours and 1 day off per week | Favors maximum shift duration of 16 hours vs. 30-hour shift with 5 hours protected sleep time | Believes that patient should be informed if resident >24 hours awake |
|---|---|---|---|---|
| Total population | 977 (81) | 952 (79) | 822 (69) | 955 (80) |
| **By political party affiliation** | | | | |
| *Democratic* | 409 (86) | 408 (85) | 342 (72) | 398 (83) |
| *Republican* | 239 (78) | 228 (75) | 204 (67) | 233 (76) |
| *Independent* | 243 (79) | 234 (76) | 197 (64) | 241 (79) |
| **By region** | | | | |
| *Northeast* | 202 (85) | 193 (81) | 154 (65) | 180 (76) |
| *Midwest* | 198 (81) | 188 (77) | 166 (68) | 195 (80) |
| *South* | 367 (80) | 366 (80) | 329 (72) | 366 (80) |
| *West* | 210 (80) | 205 (79) | 173 (66) | 214 (82) |
| **By Race** | | | | |
| *White* | 688 (81) | 661 (78) | 591 (70) | 667 (79) |
| *Black* | 98 (80) | 103 (84) | 75 (61) | 106 (87) |
| *Latino* | 119 (81) | 127 (86) | 103 (70) | 119 (81) |

Legend:

Column 1: Question read:“Thinking about ways to reduce the frequency of medical errors at hospitals, how effective do you think it would be to reduce the hours worked by medical residents at hospitals – would reducing the hours worked by medical residents be a very effective, somewhat effective, not very effective, or not at all effective in reducing the frequency of medical errors at hospitals?”

Column 2: Question read: “As you may know, doctors who work as medical residents in hospitals are required to work 80 or more hours a week, which can include shifts of up to 30 hours without sleep. Now I would like to read you a proposal that would change those work shift requirements for medical residents.. Under this proposal: -Work hours would be capped at no more than 80 hours in any single week. -Shifts would be capped at a maximum of 16 hours. -Medical residents would have at least 5 days off per month, including at least one 24 hour period per week and one 48 hour period per month. Would you favor or oppose this proposal, or are you undecided?

Column 3: Question read: “Thinking of the proposal I just read you, imagine you had two versions of the proposal. All elements would remain the same, but with one key difference. One version would say: ‘No medical resident is allowed to work more than 16 hours per shift’ and the other version would instead say ‘Medical residents' shifts are capped at 30 hours in length. During this time, they are allowed a five-hour nap, and they are not permitted to admit new patients after the first 16

hours.'"

Column 4: Question read: "Do you think patients should be informed if a medical resident who is treating them has been working for more than 24 hours?"

**Supplemental Table 3.** Support among US public for individual components of IOM resident work hours report, unweighed results.

| Questions | Support * | Oppose | Neutral | Don't know | Mean rating (95% CI) |
|---|---|---|---|---|---|
| Residents would not be allowed to work more than 16 hours per shift in patient care | 82 | 10 | 7 | 1 | 8.2 (8.0-8.3) |
| Hospitals would have to provide safe transportation home, such as a taxi or public transit ticket, for residents too fatigued to drive home safely. | 76 | 13 | 10 | 1 | 7.8 (7.6-8.0) |
| Those working a 30 hour shift would have at least 5 hours of protected sleep time between the hours of 10 pm and 8 am | 78 | 11 | 9 | 2 | 7.8 (7.7-8.0) |
| Strict rules would be established to ensure that medical residents are provided with direct, on-site supervision by more experienced doctors | 91 | 3 | 5 | 1 | 9.0 (8.7-9.1) |

| | | | | | |
|---|---|---|---|---|---|
| Work hours would be capped at no more than 80 hours in any single week | 78 | 14 | 6 | 2 | 7.9 (7.7-8.1) |
| Medical residents would have at least 5 days off per month, including at least one 24 hour period per week and one 48 hour period per month. | 86 | 6 | 7 | 1 | 8.5 (8.4-8.6) |
| Medical residents would not be allowed to moonlight, or work jobs outside of the hospital in their off hours. | 75 | 14 | 8 | 3 | 7.9 (7.7-8.1) |

* The question was answered on a scale of 0-10 where 0 is strongly oppose and 10 is strongly support and 5 is neutral. We defined “support” as 6-10, “oppose” as 0-4, “neutral” as 5.

Opening conversation:

“Hello. This is (caller name). I'm calling from National Opinion Surveys. We are conducting a public opinion survey and I would like to ask you some questions. I am not selling anything, and I will not ask for a contribution. Could I please speak with the (MALE/FEMALE) in your household, 18 years or older, who celebrated a birthday most recently?”

**Supplemental Table 4**

Question 4.1:

*Do you think patients should be informed if a medical resident who is treating them has been working for more than 24 hours?*

*[IF YES/NO ASK: Is that strongly or not so strongly YES/NO?]*

Strong yes ............................................................................... 72

[95 PERCENT CONFIDENCE INTERVAL, 70 to 75]

Not strong yes ......................................................................... 9

[95 PERCENT CONFIDENCE INTERVAL, 7 to 10]

Undecided/don’t know/refused .................................................5

[95 PERCENT CONFIDENCE INTERVAL, 4to 6]

Not strong no.............................................................................7

[95 PERCENT CONFIDENCE INTERVAL, 6 to 9]

Strong no...................................................................................7

[95 PERCENT CONFIDENCE INTERVAL, 5 to 8]

Yes ........................................................................................... 81

[95 PERCENT CONFIDENCE INTERVAL, 79 to 83]

No............................................................................................ 12

[95 PERCENT CONFIDENCE INTERVAL, 10 to 14]

Question 4.2:

*If you knew the doctor who was treating you had already been on duty for 24 hours, would you be very likely, somewhat likely, somewhat unlikely, or very unlikely to:*

*A) Feel anxious about the safety of your medical care.*

Very likely.............................................................................. 56

[95 PERCENT CONFIDENCE INTERVAL,53-59]

Somewhat likely ..................................................................... 29

[95 PERCENT CONFIDENCE INTERVAL,26-32]

Somewhat unlikely .....................................................................7

[95 PERCENT CONFIDENCE INTERVAL,5-8]

Very unlikely .............................................................................7

[95 PERCENT CONFIDENCE INTERVAL,5-8]

(Don't know) .............................................................................2

[95 PERCENT CONFIDENCE INTERVAL, 1-3]

Likely ...................................................................................... 85

[95 PERCENT CONFIDENCE INTERVAL,82-87]

Unlikely.................................................................................... 13

[95 PERCENT CONFIDENCE INTERVAL,11-15]

Question 4.2 (continued)

*B) Want to be treated by a different doctor.*

Very likely................................................................................ 51

[95 PERCENT CONFIDENCE INTERVAL,48-54]

Somewhat likely ..................................................................... 29

[95 PERCENT CONFIDENCE INTERVAL,27-32]

Somewhat unlikely .....................................................................8

[95 PERCENT CONFIDENCE INTERVAL,6-9]

Very unlikely ...............................................................................6

[95 PERCENT CONFIDENCE INTERVAL,5-8]

(Don’t know) .............................................................................6

[95 PERCENT CONFIDENCE INTERVAL,4-7]

Likely ....................................................................................... 80

[95 PERCENT CONFIDENCE INTERVAL,78-83]

Unlikely.................................................................................... 14

[95 PERCENT CONFIDENCE INTERVAL,12-16]

Question 4.2 (continued)

*C) Assume that your medical procedures and treatments will be done correctly.*

Very likely................................................................................ 25

[95 PERCENT CONFIDENCE INTERVAL,22-27]

Somewhat likely ...................................................................... 31

[95 PERCENT CONFIDENCE INTERVAL,28-34]

Somewhat unlikely .................................................................. 21

[95 PERCENT CONFIDENCE INTERVAL,18-23]

Very unlikely ........................................................................... 20

[95 PERCENT CONFIDENCE INTERVAL,17-22]

(Don’t know) .............................................................................4

[95 PERCENT CONFIDENCE INTERVAL,3-6]

Likely ...................................................................................... 55

[95 PERCENT CONFIDENCE INTERVAL,52-58]

Unlikely.................................................................................... 40

[95 PERCENT CONFIDENCE INTERVAL,37-43]
